# Exosomes: Emerging Players of Intercellular Communication in Tumor Microenvironment

**DOI:** 10.15190/d.2014.18

**Published:** 2014-09-25

**Authors:** Shrankhla Maheshwari, Anup Kumar Singh, Rakesh Kumar Arya, Deepti Pandey, Akhilesh Singh, Dipak Datta

**Affiliations:** Biochemistry Division, CSIR-Central Drug Research Institute (CDRI), Lucknow-226031, India; Academy of Scientific and Innovative Research, New Delhi, India

**Keywords:** Exosome, Tumor Microenvironment, Angiogenesis, Metastasis, Drug Resistance

## Abstract

Seminal discoveries have established the role of complex tumor microenvironment (TME) in cancer progression; and later on also uncovered that vesiculation is an integral part of intercellular communication among various cell types in coordinating the tumor assembly in a dynamic manner. Exosomes are small membrane bound endosomal vesicles, which are classically known for their role in discarding cellular wastes; however, recent reports underlined their novel role in malignancy by their release from cells into the TME. Since then, the role of exosomes have been a subject of increasing interest, as exosome mediated intercellular communications offer a novel reciprocal relationship between cancer and stromal cells within the TME and modulate the fate and function of the recipient cells to finally shape the tumor progression. Exosomes are characterised by different features including size, content and mode of delivery; and its cargo delivers interesting bioactive components in the form of proteins, miRNAs or other molecules to the target cell. In the pursuit of further study of exosomes, it was found that with the help of its distinct bioactive components, exosomes specifically regulate tumor growth, angiogenesis, metastasis as well as drug resistance properties. In fact, it acts as a bridge between different signaling networks, present inside the spatially distant cells of the heterogeneous tumor population. In the current endeavour, we have highlighted the role of exosomes in modulating the intercellular crosstalk during tumor growth and progression, and proposed certain novel roles of exosomes to address the few enigmatic questions of cancer cell biology.

## 1. Introduction

Cell secretion is a widely accepted phenomenon and a fundamental process for the maintenance of physiological functions of a cell^1^. It is accompanied mostly by microvesicles or exosome like vesicles which were previously known by many names depending on their cellular origin: ectosome, oncosome, texosome, prostasome, epididymosome and dexosome^2,3^. The concept of extracellular vesicles (EV) being introduced, in 1980s by Johnstone’s and Stahl’s groups, for secretory vesicles originating from endosome in multivesicular bodies with a function of clearing transferrin receptor during reticulocyte maturation. However, the word ‘exosome’ was proposed for the first time for EV of endosomal origin in 1987^4-6^. Exosomes are small lipid bilayer vesicles secreted by most but not all types of cells in their microenvironment. Besides their well known role in discarding waste material from the cells, exosomes play an important function in maintaining normal as well as pathological processes^7^. In case of tumor development and progression, exosome has emerged as an important mediator of cellular communication and opened up a window that extends our understanding about how certain secretory vesicles perform a critical function of transferring genetic material, induce epigenetic changes, modulate immune response to manipulate the local and systemic tumor environment to regulate cancer growth and dissemination. Current endeavour is an attempt to shed light on the emerging role of tumor-secreted exosomes as a novel player to modulate the tumor microenvironment during carcinogenesis.

## 2. Exosomes: Isolation and Characterization 

Cells secrete different kinds of vesicles in the body fluids, which vary in their biogenesis as well as in biophysical properties. Exosomes have certain characteristic features including their size (40 to 100 nm) and morphology that distinguish them from other EVs^8^. Though the role of exosomes in regulating the fate of other cells within the tissue is now widely accepted, controversies exist for the terminology in the field of EV research and they are sometimes misleading *per se* in the context of their functionality. As an attempt to address such controversies, releasing mechanism of EVs in the extracellular environment has been used as the criteria for its nomenclature. On this basis, EVs can be categorized into (i) exosomes: 40-100 nm diameter membranous vesicles of endocytic origin released by exocytic fusion of multivesicular bodies to plasma membrane, (ii) ectosomes (also referred to as shedding microvesicles): large membranous vesicles of 50-1000 nm diameter that are shed directly from the plasma membrane, and (iii) apoptotic blebs (50-5000 nm diameter): released by dying cells^9-13^. Similar to other vesicles, exosomes contain integrins, selectins but unlike others, they also harbour annexins, Rab proteins, SNAREs (v-SNARE and t-SNARE), tetraspanins like CD9, CD82, CD81, CD63 etc. and enrichment in lipids like cholesterol, sphingolipids, ceramides and glycerophospholipids with long saturated fatty acyl chains^8,14-17^.

Recent studies have shown that exosomes can be isolated *in vivo* from body fluids such as blood, urine, breast milk, amniotic fluid, malignant ascites, bronchoalveolar lavage fluid and synovial fluid etc^18-25^. These vesicles can be isolated by various techniques including differential ultracentrifugation, which can be sometimes combined with 0.1 μm to 0.22 μm filtration in order to separate the nano-sized particles from larger particles and cellular debris^26^. Immuno-affinity technique using beads against tetraspanins, CD63 or CD82, or other origin specific exosomal markers is also used to isolate these vesicles^27^. Unfortunately, the purification methods are often confusing in distinguishing exosomes from other EVs present as cross contaminations^9^. Microscopy based estimation of exosomes has its own limitation of fixation and dehydration, that may result in shrinkage and hence underestimation of actual size. However, certain modern techniques like nanoparticle based tracking analysis can be used to overcome these problems by analyzing them in PBS based on their Brownian motion in suspension to avoid shrinkage^28,29^. As conventional flow cytometers cannot differentiate between vesicles that are less than 300 nm, a novel high resolution flow cytometry–based method has been discovered for a quantitative high throughput analysis of individual (immuno-labelled) nano-sized vesicles^30-32^.

Cargo material of an exosome may include lipids, proteins (oncoproteins, tumor suppressor proteins and functional transmembrane proteins), nucleic acids (mRNA, miRNA, DNA), growth receptors, and soluble factors^33-40^. Various techniques including western blot, mass spectrometry, fluorescence activated cell sorting and immuno-electron microscopy can be used to analyse the exosomal content^41^. At a given time point, exosomes do not represent the snapshot of transcriptome of parental cells but exhibit a selective cargo profiles^42-44^. For example, exosomes from Glioma represent a different repertoire of microRNAs compared to their cells of origin, with abundance of unusual or novel non-coding RNAs of unknown functions^45^. Furthermore, it has been found that there is a low correlation between exosomal miRNA content of metastatic SKOV-3 cells versus non-metastatic OVCAR-3 cells. This dictates that specific export of miRNAs may have some potential tumor-specific modulatory role^46^.

## 3. Exosomes: Release and Uptake

**Figure 1:**
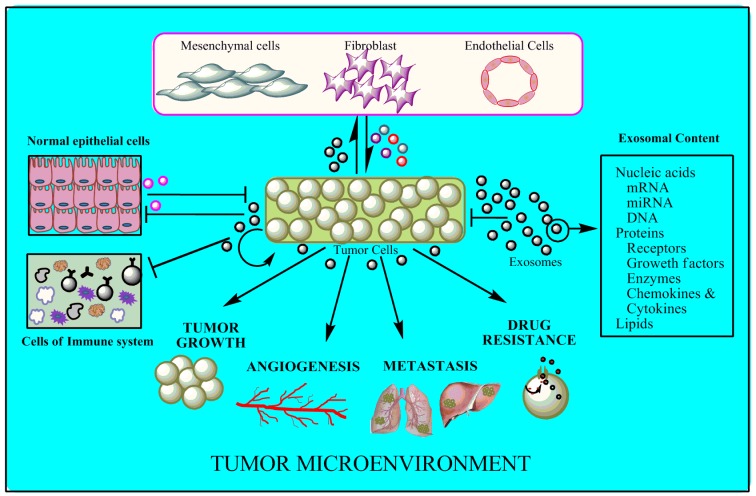
Schematic representation of exosome mediated intercellular crosstalk in tumor microenvironment

It is well established that exosomes are released by the cells after fusion of multivesicular bodies with the plasma membrane, but the molecular mechanism of their release to the microenvironment is still poorly understood^^47^^. It has been found that a number of Rab family proteins, such as Rab22a, Rab27a, Rab27b and Rab35, R-SNARE protein YKT6, glyco-sphingolipids and flotillins, as well as ceramides, are involved in the regulation of their secretion^17,48-52^. Nevertheless, the involvement of these proteins in exosome formation is dependent on cell types. Exosomal release has been found to be dependent on several physico-chemical factors like calcium, hypoxia, chemotherapeutic drug exposure, temperature and oxidative stress etc^44,53-55^ Recently, a group of scientists explored a novel feedback mechanism to control the exosomal release in a cell type specific manner. They found that exosomes from normal mammary epithelial cells at a similar concentration had a dramatic inhibitory effect on exosome production by breast cancer cells compared to exosomes from bladder cancer cells. They also proposed a dynamic equilibrium between the release and uptake of exosomes from and to the surrounding medium, depending on their concentration in extracellular environment (**Figure 1**)^56^.

The exosomal uptake is carried out by either endocytosis or phagocytosis or micro-pinocytosis^57-59^.In 2009, Parolini *et al*. demonstrated another way of exosome import to receiving cells by lipid dependant membrane fusion through "lipid rafts" of exosomal membrane. They also suggested that low pH microenvironment increases the uptake of exosomes as well as their release from the cancer cells, probably because of the intrinsically endowed negative charge due to high lipid content in exosomes. This could be the possible reason behind the cancer cells to secrete higher amount of exosomes compared to their normal counterpart and their preferential delivery to metastatic tumor cells rather than primary tumor site^60^.

## 4. Exosomes and Tumor Microenvironment (TME)

TME is composed of various cells which include stromal cells like endothelial cells, carcinoma-associated fibroblasts (CAFs), adipocytes, mesenchymal cells, immune cells (tumor-associated macrophages or TAM, T-cells etc.), as well as extracellular matrix which encircle all tumor cells, and soluble factors such as growth factors, chemokines and cytokines^61^. Besides genetic and epigenetic control, cells of the TME also possess a remodelling influence on the tumor growth and its transformation from benign to malignant. For tumor propagation, cellular communication is not only required between the tumor cells but also between cancer cells and the other neighbouring cells in TME. However, such intercellular communication can occur with or without cell-to-cell contact. Contact independent communication may take place via endocrine signaling or as an alternative; exosomes represent another way of distant cellular communication. This exosomal cross talk between resident cells imparts strong influence on inherent complexity of TME by regulating and moulding various oncogenic signaling pathways.

### 4.1 Role in Tumor Growth 

In a normal cellular homeostatic condition, cells maintain a competitive environment for deleterious cells to eradicate them from the local niche but during tumor initiation, transformed cells dominate over normal cells. Recent experimental evidence suggest an important role of exosome mediated intercellular communication in this tug of war of normal versus tumorigenic cells (**Figure 1**)^62^. As for example, exosomal miR-143 derived from non-cancerous cells have the ability to suppress the growth of cancer cells both *in vitro *and *in vivo*^63^. In contrast, breast cancer cell (MDA-MB-231, T47D:A18 and MCF-7) derived exosomes manipulate epithelial cells of the mammary duct to facilitate tumor development^64^.

During tumor growth, Wnt signaling play a pivotal role where Wnt ligands disperse spatially, however, the mechanism of their dispersal remained enigmatic. Recently, exosome mediated transfer of proteins resolve the riddle of how the hydrophobic Wnts traffic over long distances. In this context, Wnt3A has been found to be present on the surface of exosomes which explains a putative mechanism of Wnt transport to the distant recipient cells.^51^ Tumor suppressor Phosphatase and tensin homolog (PTEN) is a well established intracellular signaling molecule, but, interestingly, it can also be transported outside the cells via exosomal cargo and impart growth inhibitory function^35,65^.

Tumor derived exosomes can also influence the immune system by triggering the immunosuppressive responses, in order to favor tumor progression. These exosomes can stimulate the expansion of regulatory T-cells, which in turn impair the function of anti-tumorigenic T-cells in TME^66^. It has also been found that Transforming growth factor-beta (TGF-β) present on the surface of exosomes influences the immunosuppressive effects of regulatory T-cells^67^. Tumor derived microvesicles (MVs) also carry Fas ligands and TRAIL on their surface, which indicates their apoptosis inducing effect on activated T-cells^68,69^. Moreover, enzymes of exosomes negatively regulate T-cell activation by hydrolysis of ATP into adenosine^70^. Secretory exosomes also reduce the proliferation of Natural Killer (NK) cells or block the Interleukin 2-mediated activation of NK cells^71,72^.

### 4.2 Role in Angiogenesis

The process of angiogenesis requires a balance between pro- and anti-angiogenic factors in order to disseminate the endothelial progenitor cells to the vasculogenic site. Among the various factors, vascular endothelial growth factor (VEGF) and their receptors play a crucial role in maintaining the vascular homeostasis. During tumorigenesis, TAMs secrete VEGF, which in turn leads to vascularization to sustain tumor growth. Interestingly, microvesicle- delivery of antisense miR-150 into the mice decreased the secretion of VEGF by TAMs via targeting ING4^73^. Although the expression of EGFR on endothelial cells has been controversial, recently it has been found that exosomes facilitate a path for the expression of EGFR on the HUVEC as well as tumor associated endothelial cells, through phosphotidylserine mediated fusion between endothelial cell membrane and exosomes^74^. The process of angiogenesis also requires the activation of matrix metalloproteinases (MMPs) for the degradation of basement membrane, liberation of angiogenic factors, and sprouting of the capillaries. Tumor cells overexpress membrane bound molecule CD147, which is an extracellular MMPs inducer. Studies have shown that cancer cells (lung carcinoma, colon carcinoma, and pancreatic cancers) produce large amounts of CD147-positive MVs^75,76^ that can interact with the cells of TME and stimulate the production of MMPs. It is also observed that CD147-positive secretory vesicles of ovarian cancer cells promote angiogenic phenotype in endothelial cells (HUVECs) and pre-treatment of siRNA against CD147 suppressed their angiogenic potential^77^. Induction of endothelial cell (EC) migration and angiogenesis is mediated by the CCR1, CCL20, CXCL5, and MIF expression in ECs as evident from co-culturing them with ASTspan8 carrying exosomes^78^. Interestingly, it has also been found that D6.1A (tetraspanin Tspan8)-expressing tumor cells’ supernatant as well as D6.1A-containing exosomes, strongly induce angiogenesis^79^.

Additionally, it is also reported that hypoxic condition enhances the release of exosomes, which further promote microvascular endothelial cell migration and vasculogenesis. For example, hypoxia triggers the release of TF/VIIa bearing exosomes, which in turn increase the pro-angiogenic growth factor HB-EGF in ECs via ERK1/2-PAR2 dependent pathway^80^.

As an explanation of its capability to induce angiogenesis, exosomes has been found to modulate the many signaling pathways in a surprisingly novel mode. For instance, Notch signaling is an evolutionary conserved pathway that requires cell-to-cell contact for ligand-receptor interaction and further induction of cascade of its characteristics events. It has been shown that exosomes have signaling potential by transferring Dll-4 (ligand of notch receptor) to the neighbouring cells and incorporate it into the plasma membrane *in vitro* and *in vivo.* They have the ability to inhibit Notch signaling *in vitro* and appear to switch the endothelial cell phenotype toward tip cells phenotype, which in turn enhance vessel density^29^.

Interestingly, not every cell within the heterogenous tumor population secretes similar types of MVs, rather specific stem like cells; called cancer stem cells (CSCs) profoundly secrete specific MVs. For example, in the case of renal cancer, CD105 positive cells (CSCs) secrete MVs containing higher CD105. On co-culturing with HUVEC, only those MVs which are secreted by CSCs are able to form capillary like structures on matrigel and are able to enhance invasiveness of ECs. Furthermore, RNase pre-treatment of MVs reduced the above capabilities, which indicates that the content of these vesicles are mostly RNA molecules including miRNAs^81^. In this direction, it was further found that exogenous miR-9, previously reported to promote tumor cell motility and metastasis by repressing E-cadherin expression and increase VEGF transcription^82^, enhances EC migration and angiogenesis when exosomaly transferred to HUVECs. This exogenous miR-9 effectively reduced SOCS5 levels, leading to activation of JAK-STAT pathway^83^. Exosomes from colon cancer cells are able to induce mitosis after incorporation into endothelial cell (HUVECs) cytoplasm, which has been shown by immunostaining of both phospho-histone H3 (mitosis marker) and α-tubulin (mitotic spindle marker) in MV-treated HUVECs compared to control HUVECs^84^.

### 4.3 Role in Metastasis

Cancer metastasis involves the “leak”, or “spill” of potential cancer cells from the primary tumor, and settle down to other tissues in the body to establish a full blown secondary tumor^85^. It requires a cooperative interaction of tumor cells with non-tumor cells in the TME. Exosome mediated intercellular crosstalk plays a critical role in this coordinated process (**Figure 1**). Different tumor cells have distinct metastatic potential due to their genetic instability and exosomes are found to influence this genetic instability among tumor cells by transferring oncogenic sequences^34^. Exosomes are also found to enhance the metastatic potential of less metastatic melanoma cells B16-F1cells by transferring a metastasis marker (Met 72 tumor antigen) from highly metastatic B16 melanoma cells BL6-10^86^.

For successful evasion through extracellular matrix, tumor cells secrete MMPs or activators of MMPs like heat shock proteins (HSPs) for extracellular matrix remodelling. HSPs are also secreted through exosomes along with other proteins. It has been found that HSP90α together with annexin II in exosomes impart cell motility to their cancer cells via an interaction with an extracellular tissue plasminogen activator (tPA), which in turn activates protease plasmin^87^.Similar to extracellular matrix remodelling, vascular destabilization at the pre-metastatic niche is indispensable for cancer cell dissemination from the primary tumor site. Recently, exosomal miR-105 has been found to enhance tumor migration through enhancing vascular permeability by targeting tight junction protein ZO-1 (zonula occludens-1) in vascular endothelial cells^88^. The role of tumor derived exosomes in promoting metastatic potential of cancer cells is also supported by another elegant study in which, poorly metastasizing ASML-CD44vkd (CD44v-knockdown rat pancreatic adenocarcinoma BSp73ASML (ASMLwt) cells) cells regain metastatic capacity, when pre-treated with conditioned medium of ASMLwt cells containing exosomes. These exosomes target stromal and other cells of pre-metastatic organs to prepare metastatic niche to thrive tumor cells predominantly by transferring miRNAs (exosomal miR-494 and miR-542-3p which target cadherin-17)^89^. Furthermore Jung *et al.* also showed that conditioned media collected from ASMLwt, but not ASML-CD44vkd tumor cells promoted lymph node and lung metastasis of pancreatic cancer. Fractionation of conditioned media revealed that exosomes are the key factors which require CD44v for assembling soluble matrix^90^.

CAFs, an abundant stromal cell in the TME, support tumor growth by secreting several growth factors. They are found to stimulate breast cancer cells’ protrusive activity by exosomally transferring their endogenous Wnt11 to activate Wnt-planar cell polarity (PCP) in breast cancer cells^91^. These fibroblasts can be converted into myofibroblastic cells to support tumor growth, vascularization and metastasis. It has been shown that exosomal TGF-β was able to develop myofibroblastic phenotype in fibroblasts, through TGF-β-SMAD dependent signalling^92^. However, such differentiation was also found to occur in adipose derived mesenchymal stem cells via exosome mediated pathway in breast and ovarian cancer cells^93,94^. Recently, in an *in vitro* study, it has been demonstrated that 786‑0 renal cancer cell derived exosomes increased migration and invasion capacity of these cells by decreasing the adhesion ability and increasing the expression levels of CXCR4 and MMP‑9^95^. Moreover, metastasis promoting epithelial-mesenchymal transition (EMT) related factors, such as vimentin, hepatoma-derived growth factor (HDGF) were found in the plasma membrane and annexin 2, CK2α, and moesin in the lumen of exosomes of bladder cancer respectively, suggesting their crucial involvement in metastatic process^96^.

### 4.4 Role in Drug Resistance

In the growing area of cancer research and treatment, development of chemoresistance is a decisive challenge for chemotherapy. Decreased drug uptake, increased drug efflux, activation of detoxifying systems, activation of DNA repair mechanisms and evasion of drug-induced apoptosis etc. are several defence mechanisms, which cancer cells can develop against the chemotherapy. However, the underlying molecular mechanism for chemoresistance still remains unclear. Thus, a more efficient strategy is required to target cancer cells, which are smarter than originally believed. Genetic and molecular studies have shown that most of the malignant cancer cells have amplified multi drug resistance (*MDR*) 1 and multidrug resistance associated protein (*MRP*) genes, which are the members of ATP-binding cassette transporter (ABC transporter) superfamily^97^. Now, a series of studies dictate that tumor derived exosomes added the layer of complexity in the complex nature of cancer by transmission of resistance from resistant cells to sensitive ones (**Figure 1**). Bebawy *et al.,* showed the transmission of functional P-glycoprotein (P-gp) (MDR1) from drug resistant cancer cells (VLB_100_) to drug sensitive cancer cells (CCRF–CEM) over a co-culture period of 4 hours. They suggested that the expression of P-gp in recipient cell is not transcriptionally induced thus establishing a ‘non-genetic’ mechanism whereby MVs serve as a vector in the acquisition and spreading of MDR^98^. Recently, Wei-xian Chen *et al.* added another piece of evidence in transferring drug resistance *via* targeting MAPK pathway (putative target) through exosomal miRNAs of docetaxel resistant MCF-7 cells^99^. In a study with pulse chase and flow cytometric experimentation, vesicle shedding was represented as a potential mechanism of drug (doxorubicin) expulsion, which is found to be proportional to dose concentration^100^. Similarly, cisplatin (CDDP) was also found to expel out *via* exosomal pathway by CDDP-resistant cells, which possessed more putative CDDP transporters like ATP7A, ATP7B, and MRP2 (ABCB2) in order to escape from chemotherapeutic pressure. However, the routing of intercellular CDDP to exosomal pathway is not clear yet^101^. It has been shown that adriamycin-resistant MCF-7 cells’ derived MVs aided the resistance in recipient cells by transferring Ca^2+ ^permeable channel TrpC5, which after incorporation induced the expression of P-gp^102^.

Exosome-antibody interaction is another way by which cancer cells evade chemotherapeutic pressure. It has been found that exosome antibody sequestration reduces the antibody-dependent cytotoxicity in cancer cells by immune effector cells^103^. Moreover, HER2-overexpressing breast cancer cell lines express a full-length HER2 molecule on exosomes, which can bind to the HER2 antibody Trastuzumab to nullify its effect on tumor proliferation^104^. Thus, it seems that exosomes, besides their permissive role in tumor growth, metastasis and angiogenesis, also program the tumor cells towards resistance to chemotherapy.

## 5. Conclusion and Future Direction

Exosomes are not just cellular debris but have a functional importance in cancer biology. In a pliant TME, where every cell communicates with each other through various ways, exosomes represent a new pathway to transport the information from donor to recipient cells. As an intercellular player, exosomes exhibit their ability to promote tumor growth, metastasis niche formation and provoking angiogenesis by carrying their specific cargo. Although, extensive researches on exosomes from the past few years have revealed their various new roles in cancer progression, a deep understanding of their biogenesis, sorting, secretion and uptake are still in its infancy. Moreover, few questions also remain obscured including “how recipient cells discriminate between the exosomes they need to take from a cohort of exosomes in a microenvironmental exosomal pool”. Thus, a better understanding is warranted for selective exosomal uptake by recipient cells, in order to exert a specific stimulation.

- **Exosomes comprise of bioactive components such as proteins, RNAs, miRNAs or lipids, and play a pivotal role in modulating tumor niche by controlling intercellular crosstalk. **
- **Exosomes mediated intercellular communications and signals within TME regulate tumor growth, angiogenesis, metastasis and drug resistance.**
- **Exosomal biogenesis, sorting, secretion and discriminative uptake from the microenvironmental exosomal pool by recipient cells are emerging areas and future directions of research.**
